# Our Tears Can Help Diagnose Cancer, Parkinson’s, and Alzheimer’s

**DOI:** 10.1021/acscentsci.6c01440

**Published:** 2026-09-03

**Authors:** Marta Zaraska

## Abstract

Eye swabs, bioassays, and even smart contact lenses can “pick up” biomarkers for various conditions

## Graphical abstract

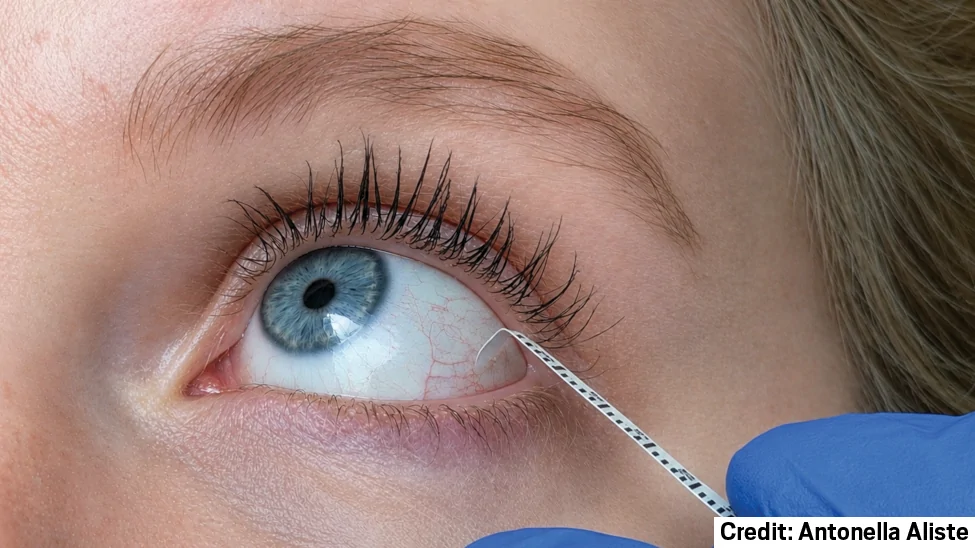

Marlies Gijs ships boxes full of tears. She packs them carefully. Tear-soaked paper strips go into small plastic tubes, and the tubes go into a box, which then gets swaddled with dry ice to make sure nothing degrades in transport.

**Figure:**
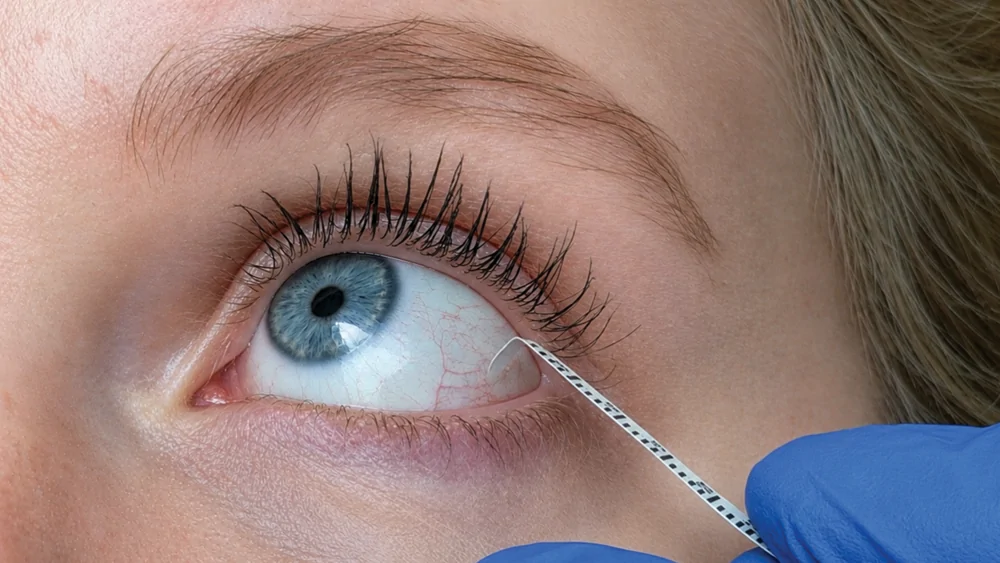
A member of Ashok Sharma’s laboratory at Augusta University dabs a subject’s eye with a paper strip. The researchers will later analyze the tears they’ve collected. Credit: Antonella Aliste.

Tear samples for medical research are hard to obtain. You cannot just order them from a biobanking company the way you can for serum samples, says Gijs, a biochemist at Maastricht University. The field is too new for that. Instead, Gijs and other scientists ship tears between laboratories, hoping to find biomarkers in tear fluid that could help doctors better diagnose diseases such as cancer, Parkinson’s disease, and Alzheimer’s disease.

A tear is far more than just salty water—it is a complex mixture of lipids, proteins, ions, and metabolites. “It contains a lot of biomarkers,” Gijs says. And when things go awry in the body, the molecular composition of tear fluid changes, a pattern that can be deciphered to diagnose disease.

Compared with blood or cerebrospinal fluid, tears are relatively free of contaminants and easily available, no needles necessary. Yet until recently, their tiny quantities have been an obstacle to studying them. According to Ashok Sharma, a computational biologist at Augusta University, scientists can usually collect no more than a few microliters, so figuring out what a tear contained “was a challenging task.”

Over the past decade, however, a plethora of new and improved technologies have emerged that make it possible to analyze molecules in tears and link them to various diseases, such as Alzheimer’s and Parkinson’s disease, breast cancer, and diabetes. Ultrasensitive technologies help researchers scan tears for disease biomarkers, while smart contact lenses laced with nanomaterials could provide continuous monitoring for various health conditions and even stress. “The advances have been phenomenal,” Sharma says.

## Scientists Share Tears and Tech

Many animals produce tears, including reptiles, birds, and fish. Humans make three kinds: emotional ones, which we shed when happy or upset; reflex tears, which may come when something irritates our eyes; and basal tears, the kind that are continually produced to lubricate and protect the eyes.

It is the basal tears that are of particular interest to researchers, since they are more predictable than other types. To collect them, scientists tend to use either capillary tubes, which are placed in the corner of the eye, or thin strips of paper, which go under the lower eyelid to absorb the fluid “a bit like kitchen paper,” Gijs says.

Back in 2005, when researchers analyzed tears extracted from such paper strips with mass spectrometry, they managed to identify 54 proteins. A decade later, new tools have helped scientists discover over 1,000 proteins in tear fluid.

Today, the tally is over 6,700—in large part thanks to improvements in mass spectrometry. Sharma believes that finding out what’s in tears is an important first step to unlocking their diagnostic potential.

**Figure:**
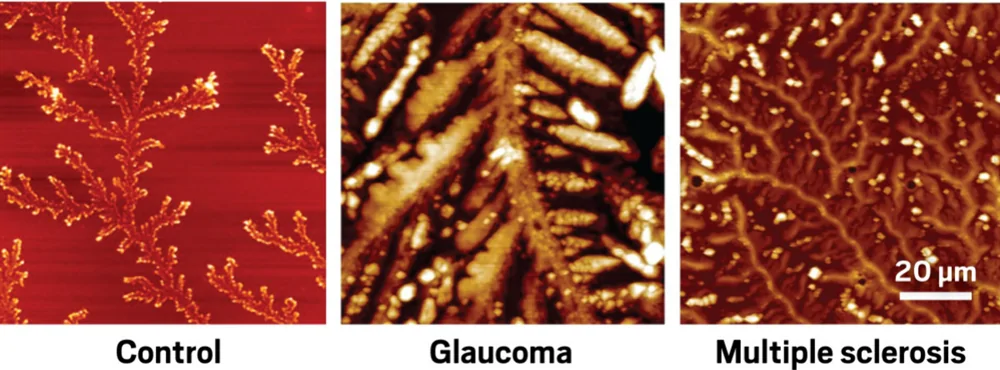
These scanning electron microscopy images show dried tear fluid from a control participant, a person with primary open-angle glaucoma, and a person with multiple sclerosis. The different characteristics of tears suggest that diagnostics based on a drop of tear fluid might be possible. Credit: Vladimír Komanický and Daria Kondrakhov.

But many new tools are expensive, so few laboratories have them. To overcome this obstacle, scientists ship tears on dry ice to those fortunate to have the technology, collaborating through Tear Research Network—a community of clinicians, scientists, and industry professionals dedicated to advancing the field of tear fluid research. Gijs and her colleagues in The Netherlands recently started work on identifying biomarkers for Alzheimer’s disease at the University of Gothenburg, in Sweden, which has the equipment to run the ultrasensitive assays they need.

The Tear Research Network is not only for shipping tears and sharing equipment; it also develops consistent research standards. Gijs realized at a 2022 conference that since the field was so new, there were no clear protocols.

Many tear researchers were using different collection and storage methods, so the results were difficult to compare between studies. Coordinating to develop standards, she realized, “would make life easier and save time, money, and effort.”

The network she now leads has over 200 professionals from across the globe. So far, they’ve issued recommendations on definitions and reporting protocols and reviewed common sample collection methods. A database of tear fluid biomarkers is in the works too. The network’s primary goal is to link tear molecules to specific diseases that have historically been difficult or invasive to diagnose, such as dementia and other neurological conditions.

## New Tools for Detecting Parkinson’s and Alzheimer’s

How brain disease biomarkers get into tears in the first place is still a bit of a mystery. For one, they might be traveling through the nerves. “The lacrimal gland—so, the main gland producing tear fluid—is innervated by nerve fibers which are very close to the brain stem,” says Paul Lingor, a neurologist at TUM University Hospital.

The biomarkers may also be seeping in through the blood, since the blood-brain barrier is often disrupted in neurodegenerative disorders. “Everything becomes a bit more leaky and more soft, so then it’s probably easier for some markers to enter the eye,” Gijs says.

In a 2021 paper, Gijs and her colleagues tested the tears of people with dementia and cognitive impairment. For the first time, they detected amyloid-β peptides and tau protein in tear fluid, molecules that are associated with neurodegeneration and make up around one-billionth of the proteins in tear fluid by mass. The researchers’ ability to identify these molecules shows that their assays are getting more sensitive and useful.

**Figure:**
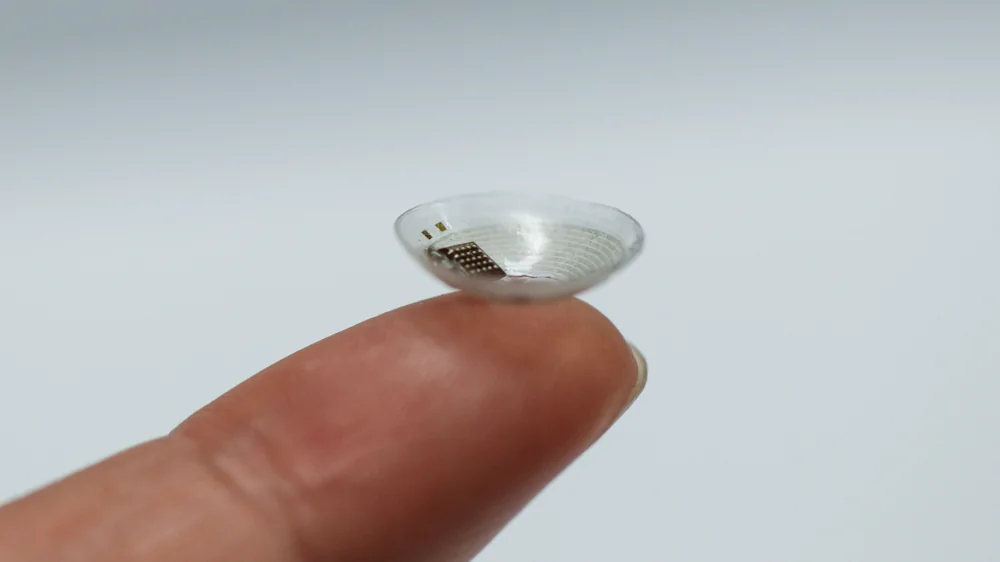
Jang-Ung Park’s laboratory at Yonsei University developed this smart contact lens for measuring stress. The graphene circuitry inside responds to cortisol in tears. Credit: Minjae Ku.

Gijs is now searching for biomarkers of neurodegenerative disorders such as Alzheimer’s disease with the help of a detection tool called nucleic acid–linked immuno-sandwich assay (NULISA). The assay is “highly sensitive, going into the attomolar range,” she says.

NULISA couples the antibody recognition capabilities of the older detection method ELISA (enzyme-linked immunosorbent assay) with more cutting-edge techniques like DNA-based barcoding and next-generation sequencing. The combination “reduces background signal and allows many proteins. . . to be measured in the same small sample,” Sharma says. Thanks to NULISA, Gijs says, she and her team are now able to detect amyloid and tau in tears ″better than ever before.″

Another way to increase sensitivity could be to grab target proteins in tear fluid and pull them out for analysis. A team at Yonsei University and Incheon National University is working on just that. Using a new immunoassay, the researchers were able to use nanoparticles to fish out adenylyl cyclase-associated protein 1 (CAP 1), a protein that the body produces to help neurons work properly, and use it as a potential biomarker for Alzheimer’s disease.

The researchers measured CAP 1 in the tears of people with no cognitive impairment, people with mild cognitive impairment, and people with Alzheimer’s. The more advanced the disease, the more CAP 1 was detected. In just 1 h, the test could correctly identify 90% of people with Alzheimer’s disease, with no false positives.

Two emerging molecular markers for mild cognitive impairment and dementia are elevated levels of the peptide glutathione and the enzyme that makes it, glutathione synthetase. Glutathione helps shield the brain from harmful molecules, and lower levels of this protective peptide have been previously detected in the blood of people with Alzheimer’s disease.

Gijs’s and Sharma’s teams published a study this April that used a new generation of Orbitrap mass spectrometry to measure the levels of glutathione synthetase and glutathione in tears. The technique achieves a higher level of mass resolution and mass accuracy than previous mass spec instruments, Sharma says. By using the new glutathione-sensing system, the researchers wrote in the paper, they could reduce the need for positron-emission tomography (PET) scans to detect dementia by 75%. That decrease would reduce the costs and exposure to radiation associated with PET scans.

Meanwhile, Lingor’s team searched tears for another hallmark protein of Parkinson’s disease, α-synuclein. Using an ultrasensitive single-molecule array (SIMOA), the researchers could pick out proteins at attomolar levels. In ELISA, the signal is measured in bulk, so very low protein levels can be hidden by background noise. But “in SIMOA, individual antibody-coated beads are isolated into tiny wells, and the instrument counts single fluorescent events,” Sharma says.

Ultimately, the technique allowed researchers to discover that people with Parkinson’s had significantly more α-synuclein in their tears than people without the disease. This finding could potentially help patients avoid lumbar punctures—procedures that are used to remove samples of cerebrospinal fluid and that may come with complications such as bleeding, pain, and infections.

While the development of high-tech diagnostics for neurological diseases will not be easy, one test based on tear proteins is already on the market. In 2022, Namida Lab, a diagnostics start-up, released its Auria test for sensing breast cancer.

You can take Auria at home: you order a kit online, collect tears with a paper strip, ship the strip back to Namida’s lab, and await your results. The test looks for two proteins linked to inflammatory processes and metastases, S100-A8 and S100-A9.

“My dream was to have something that you could pick up over the counter and be much like a pregnancy test,” says V. Suzanne Klimberg, a surgical oncologist at the University of Texas Medical Branch. She is on the clinical advisory board at Namida and is one of the authors of a 2022 Namida-funded study on which Auria is based.

Klimberg says Auria “is not as diagnostic as a mammogram because it can’t tell you where the cancer is.” Auria’s site lists the test’s false-positive rate as 42% and false-negative rate as 8%. For comparison, mammograms have false-positive and false-negative rates in the 10–15% range, according to the American Cancer Society. Klimberg hopes Auria can be an early warning that encourages more screening among people who are reluctant or unable to get mammograms.

## Wearables for Your Eyes

While tests like Auria offer a snapshot of health at a single point in time, recent technological advances allow researchers to use tears for continuous health monitoring. Enter smart contact lenses, which can gather data about molecules in tears and transmit them for analysis to a smartphone.

Wearables that fit in your eye owe their development to nanomaterials like quantum dots, graphene, and gold nanoparticles, as well as fabrication advances, such as soft lithography. More and more devices can be made tiny, see-through, and very, very sensitive. Miniaturized antennas enable real-time data transmission to external devices, while graphene and silver nanowires conduct electricity without obstructing a contact wearer’s vision.

The contact lens “is almost like a laboratory-scale device, but it has been miniaturized to the size of a postage stamp”.

Glucose is one biomarker that such smart lenses could detect. “I strongly believe that diabetes will benefit from this technology,” says Sotiria Psoma, a bionanotechnologist at the Open University. Psoma says there is now “a growing commercial interest in glucose-sensing contact lenses.”

According to Psoma, smart contact lenses could offer some advantages over continuous glucose-monitoring sensors. They would be noninvasive (no needles piercing skin) and could combine glucose sensing with vision correction as well as with monitoring for eye diseases that commonly affect people with diabetes.

A 2024 study found that glucose levels in blood are highly correlated to those in tears, and the authors reported successful tests of smart contact lenses for glucose monitoring in rabbits, dogs, and 20 humans. There was an 11-to-12 min lag time between blood glucose levels and the response in tears, which is about the same lag that continuous glucose monitors operate on, says Jang-Ung Park, a materials scientist at Yonsei University and one of the study’s authors. His team has also reported a similar smart contact lens for monitoring cholesterol.

Smart contact lenses could track the stress hormone cortisol too. If they were to appear on the market, such lenses could help monitor stress in people with high-pressure professions, like flight controllers or emergency room doctors.

Park’s team developed a lens-based cortisol sensor made of graphene, which is one atom thick and thus both flexible and extremely sensitive. “Every atom sits on the surface, where it can interact with the hormone,” Park says, so the sensor can detect minuscule amounts of cortisol.

Smart contact lenses can also use microfluidics to detect tiny amounts of biomarkers. To engineer microfluidic lenses, Ali Yetisen, a chemical engineer at Imperial College London, and his colleagues repurposed a laser that surgically corrects vision. They etched microscopic channels on the surface of contact lenses so tears could flow toward sensors.

The lens is “almost like a laboratory-scale device, but it’s been miniaturized to the size of a postage stamp,” Yetisen says. In experiments, microfluidic lenses proved useful in tracking levels of vitamin C, which is related to eye inflammation, and glutathione.

But experts admit that it is still a long way before smart contact lenses and most other tear-based diagnostics make it to the market. Besides Auria, only a few tear-fluid tests are commercially available, such as InflammaDry, for dry eye disease, while a few others are in the works, including the glucose-monitoring lenses.

“Regulatory bodies are not too familiar yet with tear fluid,” says Vladimíra Tomečková, a scientist at Pavol Jozef Šafárik University who studies the medical applications of tears. Bureaucrats, doctors, and patients alike need convincing that tear diagnostics can and do work.

Yet Gijs is optimistic that screening tears for disease biomarkers is in our future. So far, the Tear Research Network she created has been enabling new experiments, sharing new biomarkers that researchers have discovered, and publishing guidelines on how to store and ship tear samples. “I think many, many more biomarkers and tests will become available,” she says.

*Marta Zaraska is a freelance contributor to* Chemical & Engineering News *, the independent news outlet of the American Chemical Society.*

